# Integrated Transcriptomic and Targeted Metabolomic Analyses Elucidate the Molecular Mechanism Underlying Dihydromyricetin Synthesis in *Nekemias grossedentata*

**DOI:** 10.3390/plants14101561

**Published:** 2025-05-21

**Authors:** Fuwen Wu, Zhi Feng, Zhi Yao, Peiling Zhang, Yiqiang Wang, Meng Li

**Affiliations:** 1 Key Laboratory of Forestry Biotechnology of Hunan Province, Central South University of Forestry and Technology, Changsha 410004, China; 13283719075@163.com (F.W.); biotechnologyfeng@163.com (Z.F.); yaosensei@163.com (Z.Y.); 13762148137@163.com (P.Z.); 2 Yuelushan Laboratory Carbon Sinks Forests Variety Innovation Center, Changsha 410012, China

**Keywords:** *Nekemias grossedentata*, dihydromyricetin, HPLC-MS/MS, transcriptome, flavonoid 3′,5′-hydroxylase, MYB transcription factor

## Abstract

*Nekemias grossedentata* (Hand.-Mazz.) J. Wen & Z. L. Nie is a medicinal and edible plant with a high dihydromyricetin (DHM) content in its bud tips. Vine tea made from its bud tips has served as a health tea and Chinese herbal medicine for nearly 700 years. However, the molecular mechanisms underlying the high DHM content in *N. grossedentata* bud tips remain inadequately elucidated. This study conducted qualitative and quantitative analyses of bud tip flavonoids utilizing HPLC and targeted metabolomics. Core genes influencing the substantial synthesis of DHM in *N. grossedentata* were identified through integrated transcriptome and metabolome analyses. The results revealed that 65 flavonoid metabolites were detected in bud tips, with DHM as the predominant flavonoid (37.5%), followed by myricetin (0.144%) and taxifolin (0.141%). Correlation analysis revealed a significant positive correlation between *NgF3′5′H3* expression and DHM content. Co-expression analysis and qRT-PCR validation demonstrated a significant positive correlation between NgMYB71 and *NgF3′5′H3*, with consistent expression trends across three periods and four tissues. Consequently, *NgF3′5′H3* and *NgMYB71* were identified as core genes influencing the substantial synthesis of DHM in *N. grossedentata*. Elevated NgMYB71 expression in bud tips induced high *NgF3′5′H3* expression, facilitating extensive DHM synthesis in bud tips. Molecular docking analysis revealed that NgF3′5′H3 had a strong binding affinity for taxifolin. *NgF3′5′H3* was the pivotal core node gene in the dihydromyricetin biosynthesis pathway in *N. grossedentata* and was highly expressed in bud tips. The strong specific binding of NgF3′5′H3 to dihydromyricetin precursor metabolites catalyzed their conversion into DHM, resulting in higher DHM contents in bud tips than in other tissues or plants. This study aimed to elucidate the molecular mechanisms underlying the substantial synthesis of DHM in *N. grossedentata*, providing a theoretical foundation for enhancing DHM production and developing *N. grossedentata* resources.

## 1. Introduction

*Nekemias grossedentata* (Hand.-Mazz.) J. Wen & Z. L. Nie is a perennial deciduous liana belonging to the genus *Nekemias* within the family Vitaceae [1]. It is predominantly distributed in the southern Yangtze River Basin, such as Hunan, Hubei, and Guangdong provinces [2]. This plant has been utilized for nearly 700 years in Chinese medicinal herbs and health teas for its therapeutic properties [3], such as treatment of heat stroke and sore throat [4]. *N. grossedentata* is rich in bioactive compounds, notably flavonoids, polyphenols, polysaccharides, and terpenoids [5,6]. The flavonoid content is notably high, with the total flavonoid content in the bud tips reaching 45.52% [7], which is 2.4 to 4.5 times higher than that found in other flavonoid-rich plants such as *Scutellaria baicalensis* (12.30–19.34%), *Taxus chinensis* (10.66%), and *Fagopyrum cymosum* (10.34%) [8,9,10]. This high flavonoid content underscores the potential for the conservation, development, and utilization of *N. grossedentata*, earning it the designation of the “King of Flavonoids” among plants.

Dihydromyricetin (DHM) is the predominant flavonoid in *N. grossedentata*. Zeng et al. determined the DHM content in the bud tips of *N. grossedentata* using the HPLC method, finding it to be 32% [11], which constitutes 87.5% of the total flavonoid content [12]. The dihydromyricetin content in bud tips is notably higher than that in mature leaves, stems, and other tissues. Dihydromyricetin, also referred to as Ampelopsin, is classified as a dihydroflavonol. It exhibits antibacterial [13,14], antioxidant [15], anxiolytic [16], and hypoglycemic activities [17]. Furthermore, it has the capacity to delay the onset of early symptoms of Alzheimer’s disease [18], induce apoptosis in tumor cells [19], and exert other pharmacological effects. Dihydromyricetin is considered safe for drug formulation and holds significant potential for development and application as a food additive, cosmetic, and pharmaceutical.

The biosynthesis of dihydromyricetin commences through the phenylalanine pathway. The enzyme flavonoid 3′-hydroxylase (F3′H) or flavonoid 3′,5′-hydroxylase (F3′5′H) facilitates the conversion of naringenin (NAR) to eriodictyol (ERI). Subsequently, F3′5′H catalyzes the transformation of ERI into dihydrotricetin (PHF). NAR, ERI, and PHF are substrates for flavanone 3-hydroxylase (F3H), which converts them into dihydroflavonols, including dihydrokaempferol (DHK), taxifolin (DHQ), and dihydromyricetin (DHM). DHK undergoes hydroxylation at F3′H or F3′5′H to yield DHQ, which is then converted to DHM by F3′5′H. Dihydroflavonols are subsequently transformed into flavonols, including kaempferol (KAE), quercetin (QUE), and myricetin (MYE), by flavonol synthase (FLS).

Guo et al. demonstrated that CsF3′H catalyzed the conversion of NAR, DHK, and KAE into ERI, DHQ, and QUE, respectively. CsF3′5′Ha also facilitated the conversion of ERI and DHQ to DHM [20]. Mahajan et al. demonstrated that overexpression of *CsF3H* in tobacco resulted in increased flavanol content [21]. Furthermore, the overexpression of *ZmFLS1* or *BnFLS* in Arabidopsis *FLS1* mutants successfully restored the phenotypic variations initially induced by flavanol deficiency and normalized the levels of kaempferol and quercetin [22,23]. The current understanding of the molecular mechanisms underlying flavonoid synthesis in *N. grossedentata* has primarily concentrated on the analysis and identification of key enzymes involved in the entire flavonoid biosynthesis pathway [24,25]. However, the molecular regulation of dihydromyricetin, the predominant flavonoid in *N. grossedentata*, has not yet been comprehensively elucidated. It remains unclear whether the elevated expression levels of core genes upstream of dihydromyricetin synthesis in the bud tips contribute to the observed high dihydromyricetin content in this tissue. The specific molecular mechanisms responsible for this phenomenon have yet to be fully explored and warrant further investigation.

In addition to the structural genes directly involved in metabolite biosynthesis, transcription factors, such as MYB, bHLH, and WRKY, play a crucial role in influencing flavonoid metabolite synthesis [26]. This was achieved by binding to promoters, thereby regulating the expression of structural genes. For instance, the overexpression of SmMYB111 enhanced the total flavonoid content in *Salvia miltiorrhiza* Bunge [27]. The overexpression of MrMYB5 and MrMYB5L upregulated the expression of *MrF3′5′H* and *MrFLS1* [28]. DcabHLH influenced the synthesis of flavonoid metabolites by regulating *F3H* gene expression [29], and AtWRKY23 was involved in flavonol synthesis by regulating *FLS* expression [30]. Despite the recognized importance of transcription factors in dihydromyricetin synthesis in *N. grossedentata*, detailed studies on these core transcription factors remain limited. The molecular mechanisms by which transcription factors facilitate the substantial synthesis of DHM in the bud tips of *N. grossedentata* require thorough investigation.

Dihydromyricetin, a naturally occurring compound, has attracted considerable attention due to its diverse therapeutic properties. Current research predominantly aimed to elucidate its pathological roles and pharmacodynamic mechanisms, including structural modification [31] and drug development [32]. However, the molecular mechanisms underlying the notably high DHM content in the bud tips of *N. grossedentata* remain insufficiently explored and documented. Consequently, this study utilized bud tips from *N. grossedentata*, which are rich in DHM, and combined transcriptome and metabolome analyses to identify the core structural genes and transcription factors influencing high DHM content in bud tips, based on differential gene expression analysis, co-expression network analysis, qRT-PCR validation, and molecular docking simulation. This study aimed to elucidate the molecular mechanisms responsible for the extremely high DHM content in the bud tips of *N. grossedentata*. Additionally, it sought to provide a significant reference for enhancing DHM production from its biosynthetic sources and for the development and utilization of *Nekemias grossedentata*.

## 2. Results

### 2.1. Determination and Analysis of Total Flavonoids and Dihydromyricetin Content in Bud Tips of Nekemias grossedentata

**Figure 1:**
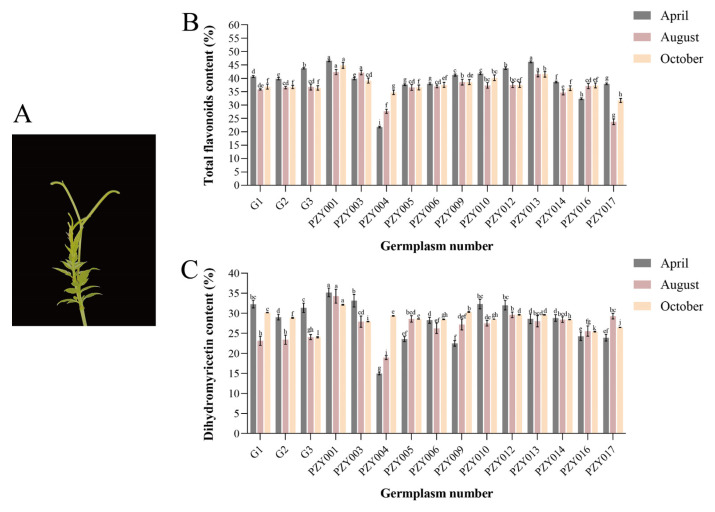
Analysis of total flavonoid and dihydromyricetin content in bud tips of 15 germplasms across April, August, and October. (**A**) Bud tip of *Nekemias grossedentata*. (**B**) Total flavonoid content. (**C**) Dihydromyricetin content. Standard deviations are indicated by error bars, and the letters in the graphs denote significant differences (*p* < 0.05) in content among germplasms within the same period.

To quantify the total flavonoid and dihydromyricetin (DHM) content in the bud tips of *Nekemias grossedentata*, this study investigated the total flavonoid and dihydromyricetin content in the bud tips of 15 germplasms during April, August, and October, employing spectrophotometric and HPLC methodologies (Figure 1A). The data indicated that the total flavonoid content in the April bud tips of the PZY004 germplasm was at a lower level of 21.81% among all the germplasms. In contrast, the total flavonoid content in the April bud tips of the PZY001 germplasm was significantly higher at 46.59%, 2.14 times that of the PZY004 germplasm (Figure 1B). In terms of temporal variation, the total flavonoid content was higher in bud tips grown in April. Conversely, the total flavonoid content in bud tips grown in August and October was on average lower than that in bud tips grown in April.

The data indicated that the dihydromyricetin content in the April bud tips of the PZY004 germplasm was at a lower level of 14.98% among all the germplasms. In contrast, the dihydromyricetin content in the April bud tips of the PZY001 germplasm was significantly higher at 35.18%, 2.35 times that of the PZY004 germplasm (Figure 1C). The proportion of DHM relative to the total flavonoid content in various bud tip samples ranged from 52.58% to 89.67%.

The comparative analysis identified significant variations in the total flavonoid and dihydromyricetin content within the bud tips of the germplasms PZY001 and PZY004. Consequently, the bud tips of these two germplasms were selected for targeted flavonoid metabolome analysis. This selection facilitated further investigation into the molecular mechanisms underlying the substantial synthesis of dihydromyricetin in the bud tips of *N. grossedentata*.

### 2.2. Qualitative and Quantitative Analysis of Flavonoids in the Bud Tips of Nekemias grossedentata

To elucidate the flavonoid composition and content in the bud tips of *Nekemias grossedentata*, this study selected the bud tips of germplasms PZY001 and PZY004 in April, August, and October for UPLC-MS/MS analysis. Each group was designated as follows: PZY001: ST-1-4, ST-1-8, ST-1-10; PZY004: ST-4-4, ST-4-8, ST-4-10, with four biological replicates established for each group.

**Figure 2:**
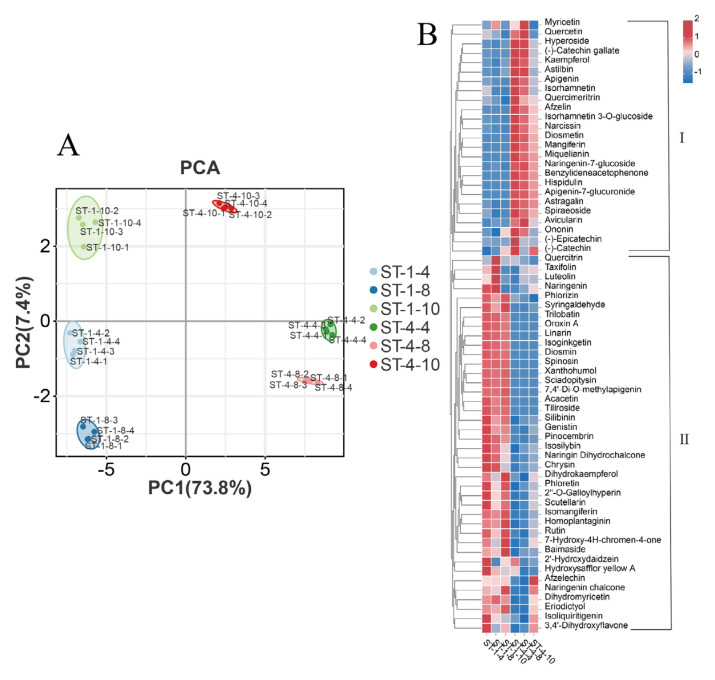
Targeted metabolomic analyses. (**A**) Principal component analysis of the six bud tip sample groups. (**B**) Heatmap of the overall clustering of 65 flavonoids. ST-1-4: PZY001 germplasm bud tips in April; ST-1-8: PZY001 germplasm bud tips in August; ST-1-10: PZY001 germplasm bud tips in October. ST-4-4: PZY004 germplasm bud tips in April; ST-4-8: PZY004 germplasm bud tips in August; ST-4-10: PZY004 germplasm bud tips in October. The flavonoids categorized within Clusters I and II exhibited distinct patterns in their content across the various samples.

A preliminary analysis of the variations in flavonoid content among the six groups was performed using Principal Component Analysis (PCA). The observed dispersion among the six groups was substantial, indicating significant differences in flavonoid content (Figure 2A). Considerable variations were observed in the types and contents of flavonoids present in bud tips of different germplasms and periods. The employed detection method demonstrated stability, and the data quality was deemed reliable, making it suitable for subsequent analyses. The OPLS-DA analysis performed on the six-group sample revealed that the R^2^X, R^2^Y, and Q^2^ values surpassed 0.9 across the nine comparison groups, thereby demonstrating the model’s feasibility. During the verification of the ranking test for the OPLS-DA statistical model, all predicted values were lower than the actual R^2^ and Q^2^ values of the original model. Additionally, the intercept of the Q^2^ regression line was less than 0.05. As the horizontal coordinate replacement retention decreased, both R^2^ and Q^2^ decreased, and the regression line exhibited an upward trend. The results indicated that the replacement test models were all valid, there was no overfitting, and the constructed discrepancy model was reliable.

Qualitative analysis revealed that a total of 65 flavonoids were identified using UPLC-MS/MS across six groups of bud tips. The detected flavonoids comprised 10 flavones, 10 flavonoid glycosides, 7 flavonol glycosides, 6 dihydroflavonols, 6 flavonols, 5 chalcones, 4 flavanols, 3 flavanones, 3 isoflavones, 3 dihydrochalcones, 2 biflavonoids, 2 phenolic acids, 1 flavonone, 1 flavonone glycoside, 1 chalcone glycoside, and 1 C-glycosyl flavonoid (Table S1). Quantitative analysis revealed that dihydromyricetin was the predominant flavonoid compound across all bud tip sample groups, reaching 37.5%. This was followed by myricetin (MYE, 0.144%) and taxifolin (DHQ, 0.141%).

The 65 flavonoids were clustered and visualized to illustrate the content of the various flavonoid compounds (Figure 2B). The flavonoid compounds were categorized into two clusters based on their expression patterns. Cluster II comprised a total of 40 flavonoids, including DHM and various upstream precursor metabolites such as taxifolin, with their content being significantly higher in the bud tips of the PZY001 germplasm across all three periods (ST-1-4, ST-1-8, and ST-1-10) compared to the bud tips of the PZY004 germplasm during the same periods (ST-4-4, ST-4-8, and ST-4-10). Conversely, Cluster I comprised 25 flavonoids, including a range of downstream metabolites of DHM, such as myricetin, which were significantly more abundant in ST-4-4, ST-4-8, and ST-4-10 than in ST-1-4, ST-1-8, and ST-1-10. Significant variations were observed in the total flavonoid content across different *Nekemias grossedentata* germplasms. Notably, there were substantial differences in dihydromyricetin, the primary flavonoid metabolite, as well as distinct patterns in the content and type of the remaining multiple flavonoids.

### 2.3. Analysis of Significant Differential Metabolites

To gain a more precise understanding of the differences in flavonoids present in the bud tips of each group, the variable importance in projection (VIP) values obtained from OPLS-DA analysis, along with the *p*-values derived from univariate statistical *t*-tests, exhibiting VIP ≥ 1 and *p* < 0.05, were selected as the criteria to identify significant differential metabolites between the various groups. Five metabolites exhibited significant differences between the comparison groups: dihydromyricetin, taxifolin, naringenin, quercitrin, and hyperoside. The dihydromyricetin content was upregulated in the ST-4-4-vs-ST-4-8, ST-4-4-vs-ST-4-10, and ST-4-8-vs-ST-4-10 comparative groups, indicating a significant incremental trend in dihydromyricetin content in the bud tips of the PZY004 germplasm over various periods (Table S2).

### 2.4. Transcriptome Analysis of Nekemias grossedentata Bud Tips Across Distinct Periods

Quantitative analysis revealed that the dihydromyricetin content in the bud tips of the PZY001 germplasm remained relatively stable across April, August, and October, with contents of 35.4%, 37.5%, and 35.3%, respectively. In contrast, the dihydromyricetin content in the bud tips of the PZY004 germplasm exhibited a significant upward trend from April to October. Specifically, the dihydromyricetin content was 18.69% in April and increased to 32.18% in October, representing 1.72 times compared to April. These results indicated significant temporal variations in dihydromyricetin content within the bud tips of the PZY004 germplasm. Therefore, bud tips of the PZY004 germplasm, which exhibited significant variations in dihydromyricetin content across three distinct periods, were selected for eukaryotic parametric transcriptome sequencing and analyses. This approach facilitated the identification and examination of differentially expressed genes to elucidate the molecular mechanisms underlying the substantial synthesis of dihydromyricetin in the bud tips of *Nekemias grossedentata*. Bud tip samples from the three periods were designated ST-4-4, ST-4-8, and ST-4-10, with four biological replicates established for each group (Tables S3 and S4).

**Figure 3:**
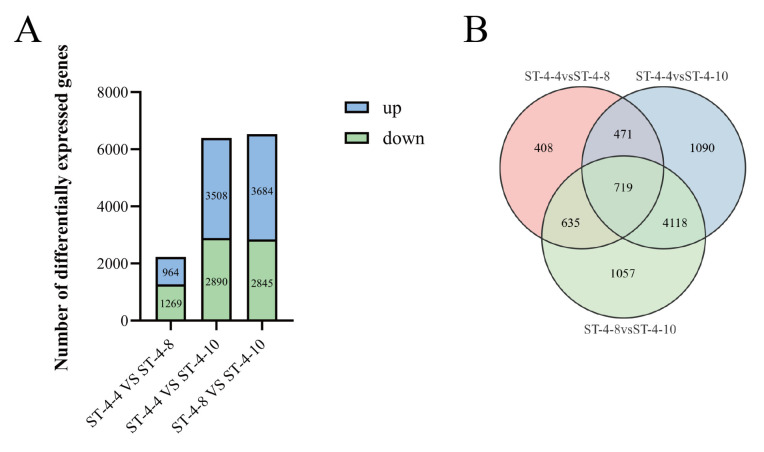
Analysis of differentially expressed genes in the bud tips of *Nekemias grossedentata* across three periods. (**A**) The number of differentially expressed genes. (**B**) Venn diagram of the number of differentially expressed genes among the various comparison groups.

There were 2233 (964 upregulated and 1269 downregulated), 6398 (3508 upregulated and 2890 downregulated), and 6529 (3684 upregulated and 2845 downregulated) differentially expressed genes (DEGs) identified from ST-4-4 VS ST-4-8, ST-4-4 VS ST-4-10, and ST-4-8 VS ST-4-10, respectively (Figure 3A). The integration of quantitative assay results for DHM revealed that a more pronounced difference in DHM content corresponded to a greater number of DEGs in the comparisons between ST-4-4 VS ST-4-10 and ST-4-8 VS ST-4-10. A total of 5943 DEGs were identified across the comparison groups (Figure 3B).

Through the application of trend clustering analysis, the 5943 DEGs screened were categorized into eight distinct profiles based on their expression patterns across the three periods of the bud tips (Figure S1). The expression levels of DEGs in profiles 3, 4, and 7 exhibited significant differential changes across the three periods. The DEGs in profile 4 exhibited stabilization of expression levels from ST-4-4 to ST-4-8, followed by a significant increase from ST-4-8 to ST-4-10. Conversely, the DEGs in profile 7 demonstrated a consistent increase in expression from ST-4-4 to ST-4-10. The dihydromyricetin contents at ST-4-4, ST-4-8, and ST-4-10 were 18.69%, 20.18%, and 32.18%, respectively. The expression patterns of the DEGs in profiles 4 and 7 were similar to the changes in dihydromyricetin content over the same period. Consequently, it was judged that profiles 4 and 7 contained candidate genes that played a role in the positive regulation of dihydromyricetin synthesis.

However, the expression levels of DEGs in profile 3 tended to stabilize from ST-4-4 to ST-4-8, followed by an overall significant decrease in expression from ST-4-8 to ST-4-10. Concurrently, the dihydromyricetin contents at ST-4-4, ST-4-8, and ST-4-10 were 18.69%, 20.18%, and 32.18%. Notably, the expression pattern of DEGs in profile 3 was inversely related to the changes in dihydromyricetin content during the same period. Consequently, it was judged that profile 3 contained candidate genes involved in the negative regulation of dihydromyricetin synthesis. The DEGs in profiles 3, 4, and 7 were proposed as potential candidate genes influencing dihydromyricetin content.

Functional annotation and enrichment analyses were conducted to better understand the biological processes, metabolic pathways, and signaling pathways related to the DEGs in profiles 3, 4, and 7. The results showed that the DEGs in profiles 3, 4, and 7 were mainly annotated for functions such as metabolic processes, catalytic activity, and binding (Figure S2), and were predominantly enriched in pathways related to environmental adaptation and the biosynthesis of other secondary metabolites (Figure S3).

### 2.5. Screening, Identification, and Analysis of Candidate Genes in the Dihydromyricetin Synthesis Pathway

To elucidate the molecular mechanisms responsible for the notably high content of dihydromyricetin in the bud tips of *Nekemias grossedentata*, based on the functional annotation information of the genes in the transcriptome data, the relevant genes located in the pathway of dihydromyricetin synthesis were found in profiles 3, 4, and 7, including *F3H*, *F3′5′H*, and *FLS*. Subsequent gene identification analysis revealed one flavanone 3-hydroxylase gene (*F3H*), two flavonoid 3′, 5′-hydroxylase genes (*F3′5′H*), and three flavonol synthase genes (*FLS*). These genes were designated according to their respective chromosomal positions (Table 1).

### 2.6. Combined Analysis and Screening of Candidate Core Structural Genes Influencing Dihydromyricetin Synthesis

**Figure 4:**
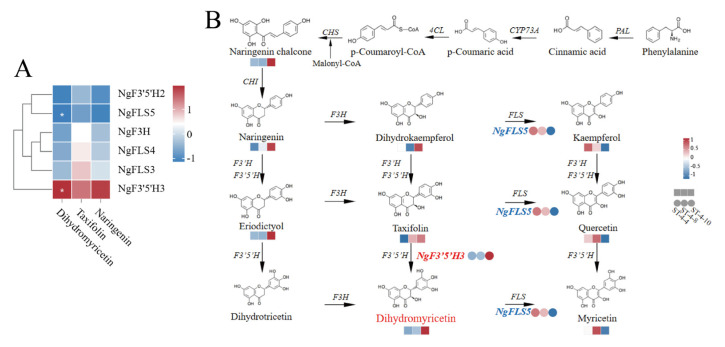
Correlation analysis was used to examine the relationship between the expression levels of candidate structural genes and the differential metabolite content. (**A**) The heatmap illustrates the correlation between the three differential metabolite contents and the expression levels of the six candidate structural genes. * denotes a statistically significant difference, with 0.01 < *p* < 0.05. (**B**) Pathways of various genes involved in dihydromyricetin biosynthesis are depicted, with upregulated genes highlighted in red, downregulated genes in blue, metabolite abundance represented by squares, and gene expression levels indicated by circles.

To further elucidate the core structural genes responsible for the elevated dihydromyricetin content in the bud tips of *Nekemias grossedentata*, Pearson’s correlation coefficient analysis was performed to investigate the relationship between the expression levels of the six candidate structural genes and the content of the three differential metabolites: dihydromyricetin, taxifolin, and naringenin. The correlation was visualized in the form of a clustered heatmap. The results showed a significant positive correlation between *NgF3′5′H3* and dihydromyricetin content (0.999, *p* < 0.05) and a significant negative correlation between *NgFLS5* and dihydromyricetin content (0.997, *p* < 0.05) (Figure 4A, Table S5).

In the dihydromyricetin biosynthesis pathway, the enzyme F3H is situated upstream of dihydromyricetin synthesis and is responsible for producing dihydroflavonols, including dihydrokaempferol, taxifolin, and dihydromyricetin, by catalyzing the 3′ position on the central ring of flavanones such as naringenin, eriodictyol, and dihydrotricetin. Similarly, F3′5′H is located upstream of DHM synthesis and facilitates the production of dihydromyricetin by catalyzing taxifolin. In contrast, FLS is positioned downstream of dihydroflavonols and plays a regulatory role in flavonol synthesis, including kaempferol, quercetin, and myricetin (Figure 4B). The expression level of *NgF3′5′H3* gradually increased from ST-4-4 to ST-4-10. This pattern was consistent with the trend observed for dihydromyricetin content, and a significant positive correlation was identified between the expression of *NgF3′5′H3* and dihydromyricetin content. Nonetheless, no correlation was observed between the expression level of *NgF3H*, which is situated upstream of the dihydromyricetin synthesis pathway, and dihydromyricetin content. Therefore, *NgF3′5′H3* was identified as a pivotal node gene in the dihydromyricetin synthesis pathway in the bud tips of *N. grossedentata*. The upregulation of *NgF3′5′H3* promoted the conversion of precursor substances into dihydromyricetin.

The expression of *NgFLS5* was significantly and inversely correlated with the dihydromyricetin content. Specifically, the expression levels of *NgFLS5* were elevated during the ST-4-4 and ST-4-8, followed by a pronounced decline during the ST-4-10. This pattern was inversely related to the trend observed for dihydromyricetin content but corresponded with the changes in flavonol levels (kaempferol, quercetin, and myricetin). The *NgF3′5′H3* and *NgFLS5* genes were identified as candidate core structural genes influencing dihydromyricetin synthesis in *N. grossedentata* bud tips.

### 2.7. Screening and Expression Validation of Core Transcription Factors Regulating Dihydromyricetin Synthesis

**Figure 5:**
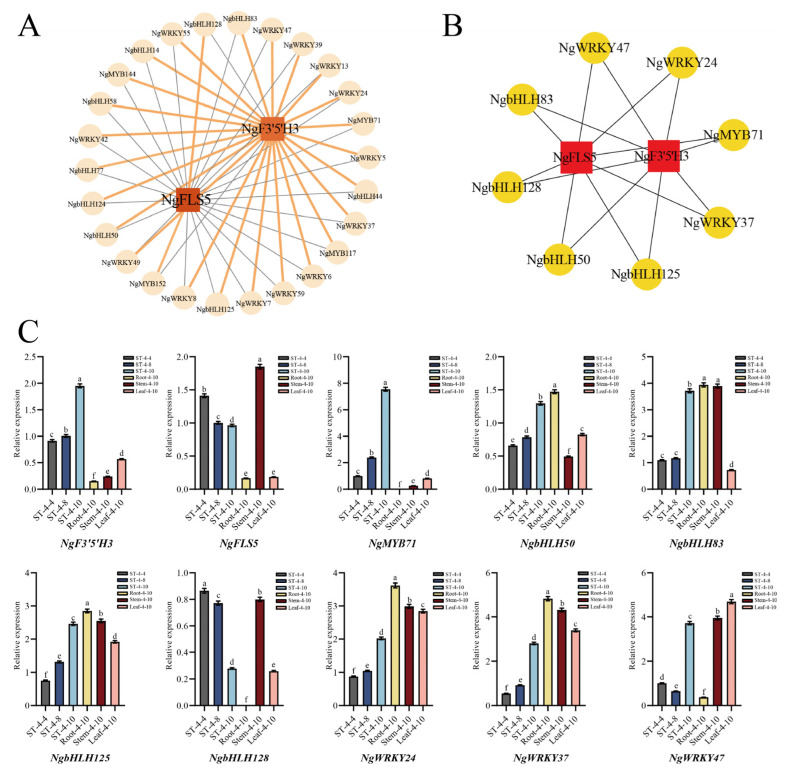
Screening of core transcription factors regulating dihydromyricetin synthesis. (**A**) Co-expression regulatory network of candidate core structural genes and TFs. (**B**) Analysis of candidate gene co-expression. (**C**) Validation of genes expression patterns in bud tips and various tissues at different periods using qRT-PCR. The different letters in (**C**) indicate significant differences at the *p* < 0.05 level.

Transcription factors (TFs), such as MYB, bHLH, and WRKY, were identified across various plant species for their capacity to either activate or repress the expression of structural genes within the flavonoid synthesis pathway. This regulatory mechanism indirectly influenced flavonoid synthesis and served as the primary driving factor affecting flavonoid production. In this study, the MYB, bHLH, and WRKY family transcription factors in profiles 3, 4, and 7 were selected for correlation analysis with two candidate core structural genes. The correlation was visualized through a co-expression network (Figure 5A), which further screened out eight candidate transcription factor genes exhibiting a high correlation with the candidate core structural genes: *NgMYB71* (*Chr02.g02925*), *NgbHLH50* (*Chr07.g09557*), *NgbHLH83* (*Chr11.g15731*), *NgbHLH125* (*Chr20.g25640*), *NgbHLH128* (*Chr20.g25841*), *NgWRKY24* (*Chr09.g13469*), *NgWRKY37* (*Chr13.g17873*), and *NgWRKY47* (*Chr18.g23294*) (Figure 5B). These eight candidate transcription factors were believed to play a role in regulating the synthesis and accumulation of dihydromyricetin in the bud tips of *N. grossedentata* by modulating the expression of the candidate core structural genes *NgF3′5′H3* and *NgFLS5*.

The expression patterns of *NgF3′5′H3* and *NgFLS5*, along with eight candidate transcription factor genes, were detected and analyzed using quantitative real-time PCR (qRT-PCR) in the bud tips of the PZY004 germplasm in April, August, and October. Additionally, these analyses were conducted on roots (Root-4-10), stems (Stem-4-10), mature leaves (Leaf-4-10), and bud tips (ST-4-10) in October of the PZY004 germplasm. The findings indicated that among the eight candidate transcription factors, only NgMYB71 exhibited an overall consistent expression pattern with *NgF3′5′H*. The expression levels progressively increased from ST-4-4 to ST-4-10, with continued elevation observed in Root-4-10, Stem-4-10, Leaf-4-10, and ST-4-10 samples (Figure 5C). A correlation analysis was performed to examine the expression levels of two candidate core structural genes, *NgF3′5′H3* and *NgFLS5*, along with eight candidate transcription factors in bud tips across various periods and different tissue parts at the same period. The results showed that only the NgMYB71 transcription factor exhibited a highly significant positive correlation with *NgF3′5′H3* expression (*p* < 0.01) (Table 2). As a result, *NgF3′5′H3* and *NgMYB71* were identified as the core genes influencing the substantial synthesis of dihydromyricetin in the bud tips of *N. grossedentata*. These genes were highly expressed in bud tips, facilitating the extensive production of dihydromyricetin in bud tips.

The qRT-PCR results revealed a progressive increase in *NgF3′5′H* expression from ST-4-4 to ST-4-10, whereas *NgFLS5* expression continuously declined during the same period. These qRT-PCR results were consistent with the RNA sequencing (RNA-seq) results, corroborating the reliability of the data.

### 2.8. Bioinformatics Analysis of NgF3′5′H3

Utilizing genome and transcriptome data, the conserved domain of NgF3′5′H was analyzed using NCBI CD-Search and InterPro (Figure S4). These findings indicated that *NgF3′5′H* was a member of the *CYP450* superfamily and possessed the conserved domain of flavonoid 3′, 5′-hydroxylase (PLN00110). The complete CDS length of *NgF3′5′H3* was 1527 bp, encoding 508 amino acids. Predictive analysis indicated that the protein did not contain a signal peptide or transmembrane sequence, indicating that it was unlikely to be a secreted or membrane-associated protein. Furthermore, secondary structure prediction revealed that α-helix and irregular coiling were the predominant structural elements of the NgF3′5′H3 protein.

**Figure 6:**
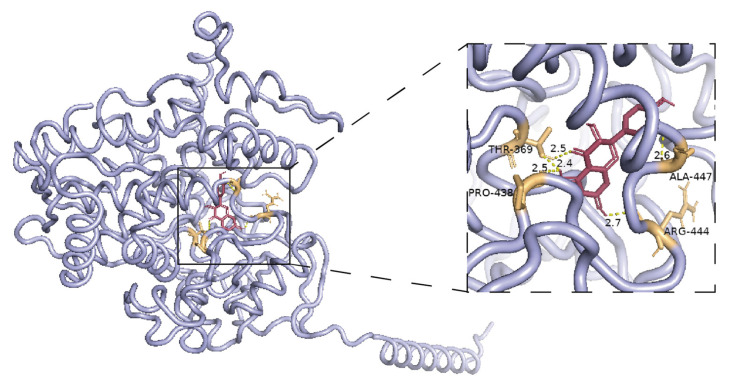
Predictive analysis of the binding site of NgF3′5′H3 protein with taxifolin. The pink structure represents taxifolin, the blue structure denotes the NgF3′5′H3 protein, and the yellow dashed lines illustrate the hydrogen bonding interactions.

To elucidate the homology of *NgF3′5′H3*, a phylogenetic analysis was performed by comparing this gene with *F3′5′H* genes from various species. The results indicated that *NgF3′5′H3* is homologous to the *F3′5′H* genes found in *Vitis riparia*, *Vitis vinifera*, *Vitis amurensis*, and *Vitis davidii* (Figure S5). The amino acid sequence of NgF3′5′H3 was compared with that of F3′5′H from related species to predict its catalytic activity. The analysis revealed that the substrate recognition site 6 (SRS6) of NgF3′5′H3 contained a conserved alanine residue (Ala). Consequently, it was hypothesized that NgF3′5′H3 possesses a typical F3′5′H catalytic function (Figure S6). Molecular docking results indicated that the binding energy between the NgF3′5′H3 protein and taxifolin, a precursor metabolite of dihydromyricetin, was −7.4 kcal/mol, indicating an exceptionally strong binding affinity (Figure 6). Additionally, several MYB recognition and binding sites were predicted in the promoter region of *NgF3′5′H* (Figure S7).

Expression pattern analysis of the core gene *NgF3′5′H3* indicated that *NgF3′5′H3* is a crucial node gene in the dihydromyricetin biosynthesis pathway within the bud tips of *Nekemias grossedentata*. It can be inferred that NgF3′5′H3 specifically interacted with dihydromyricetin precursor metabolites, catalyzing their transformation into dihydromyricetin. The elevated expression of the NgMYB71 transcriptional regulator in bud tips induced a significant increase in *NgF3′5′H3* expression within the same tissue, thereby facilitating the substantial conversion of taxifolin to dihydromyricetin in *Nekemias grossedentata*.

## 3. Discussion

### 3.1. Analysis of Flavonoids in Bud Tips of Nekemias grossedentata

*Nekemias grossedentata* is often regarded as the “King of Flavonoids” in the botanical realm—the total flavonoid content can comprise up to 45% in bud tips [7]. Despite this significant presence, there is a lack of research focusing on the characterization and quantification of flavonoids in *Nekemias grossedentata*. Dihydromyricetin (DHM) is a dihydroflavonol identified in various species, such as *Hovenia dulcis Thunb* (approximately 1.388%) [33], poplar flowers (0.049%) [34], and *Cedrus deodara* (0.013%) [35]. Notably, it was detected at the highest content in the bud tips of *Nekemias grossedentata*.

In this study, a total of 65 flavonoids were detected in the bud tips of *N. grossedentata* through qualitative and quantitative analyses utilizing HPLC-MS/MS technology. Dihydromyricetin was the predominant flavonoid compound across several bud tip groups, with a content of up to 37.5%. Myricetin was the second most prevalent flavonoid compound, with a content of 0.144%. Taxifolin and quercitrin were the third and fourth most prevalent flavonoid compounds, with contents of up to 0.141% and 0.043%, respectively. He et al. merely conducted a qualitative analysis of the compounds present in the stems and mature leaves of *N. grossedentata*, identifying 67 flavonoids using UPLC-Q-TOF-MS/MS technology [36]. Untargeted metabolomics offers extensive coverage of detected compounds, whereas targeted metabolomics facilitates the absolute quantitative analysis of metabolites but is limited in coverage [37]. This disparity resulted in variations in the flavonoids identified and detected in *N. grossedentata* across different studies. Zeng et al. measured that the content of dihydromyricetin in *N. grossedentata* bud tips could reach 32% using HPLC [11]. Zhang et al. found that the content of myricetin in wild *N. grossedentata* bud tips was approximately 0.116% by HPLC [38], thereby corroborating the reliability of the data obtained from this assay.

Qualitative and quantitative analyses of the targeted flavonoid metabolome revealed that the contents of dihydromyricetin and its upstream metabolites, including naringenin, eriodictyol, dihydrokaempferol, and taxifolin, were elevated in the bud tips of PZY001 compared to those of PZY004. The naringenin, eriodictyol, dihydrokaempferol, taxifolin, and dihydromyricetin contents in the bud tips of PZY001 were 1.57, 1.74, 1.29, 1.27, and 1.52 times higher than those in the bud tips of PZY004, respectively. However, the contents of flavonols downstream of dihydroflavonols, including kaempferol, quercetin, and myricetin, were elevated in the bud tips of PZY004 compared to those of PZY001. The contents of kaempferol, quercetin, and myricetin in the bud tips of PZY004 were 1.82, 1.53, and 1.11 times higher than those in the bud tips of PZY001, respectively. The findings indicated that the content of dihydromyricetin, the predominant flavonoid in *Nekemias grossedentata*, exhibited significant variation across different germplasms. In addition, the composition and accumulation patterns of other flavonoids exhibited distinct variations. It was suggested that germplasm is a crucial factor influencing the quality of *Nekemias grossedentata* resources.

### 3.2. Identification and Validation of Core Genes Influencing Dihydromyricetin Contents in Bud Tips

F3′5′H, recognized as a critical rate-limiting enzyme in the flavonoid biosynthesis pathway, has been confirmed to enhance flavonoid production in plants [20,39,40]. This study identified the core genes involved in dihydromyricetin synthesis by selecting dihydromyricetin-rich bud tips as materials and employing a combined transcriptome and metabolome analysis. The results revealed that only the expression level of *NgF3′5′H3* was significantly and positively correlated with dihydromyricetin content. The expression level of *NgF3′5′H3* increased sequentially in ST-4-4, ST-4-8, and ST-4-10, aligning with the trend of dihydromyricetin content in the bud tip samples across the three periods. The qRT-PCR results indicated that the expression level of *NgF3′5′H3* increased sequentially in the roots, stems, mature leaves, and bud tips in October of *N. grossedentata*, which corresponded with the trend of changes in dihydromyricetin. The highest DHM content was observed in the bud tips of *N. grossedentata*, surpassing that found in mature leaves, stems, and roots [41]. The expression level of *NgFLS5* exhibited a negative correlation with the dihydromyricetin content. Therefore, this study hypothesized that the downregulation of *NgFLS5* expression facilitates the accumulation of dihydromyricetin and other dihydroflavonol compounds.

Analysis of the substrate recognition site (SRS) sequence of NgF3′5′H3 facilitated a deeper understanding of its catalytic function. SRS6 is a critical region of CYP450 enzymes that is essential for substrate recognition. Most experimental evidences indicate that the SRS6 of the F3′5′H protein typically contains a conserved alanine residue (Ala) [42]. This study revealed that the SRS6 substrate recognition site of NgF3′5′H3 protein contained a conserved alanine residue (Ala). This observation aligned with the results of the sequence and functional analyses of the SbF3′5′H protein conducted by Pei et al. [43]. These findings not only suggested that NgF3′5′H3 exhibited a typical F3′5′H protein catalytic function but also implied that it may possess a catalytic function similar to that of the SbF3′5′H protein in *N. grossedentata*, facilitating the conversion of dihydrokaempferol and taxifolin to dihydromyricetin.

Transcription factors are the primary driving factors in the biosynthesis of flavonoids. They have been demonstrated to regulate the expression of *F3′5′H* in numerous plant species, thereby influencing flavonoid content [44,45,46]. In this study, co-expression analysis and qRT-PCR revealed that only NgMYB71 exhibited a highly significant positive correlation with the expression level of *NgF3′5′H3*. Furthermore, the expression patterns of these two genes were consistent in bud tips across different periods and in various tissues at the same period. The promoter region of *NgF3′5′H3* was predicted to contain multiple recognition and binding sites for MYB transcription factors. *NgF3′5′H3* and *NgMYB71* were identified as the core genes influencing the substantial synthesis of dihydromyricetin in the bud tips of *N. grossedentata*. The elevated expression level of NgMYB71 in the bud tips subsequently induced a high expression level of *NgF3′5′H3* in the same tissue, resulting in a significantly greater content of dihydromyricetin in the bud tips than in other tissues.

The molecular docking results further demonstrated a significant binding activity between the NgF3′5′H3 protein and taxifolin, a precursor in the synthesis of dihydromyricetin, with a binding energy of −7.4 kcal/mol. It is widely recognized that binding energy below 0 kcal/mol suggests the potential for spontaneous binding between the protein and the small-molecule ligand. Furthermore, a binding energy below −5 kcal/mol indicates favorable binding activity, whereas a binding energy below −7 kcal/mol signifies strong docking activity between the protein and the small-molecule ligand [47]. Wang and XI et al. employed molecular modeling techniques to predict the strong binding affinity between the target protein and the target metabolite prior to conducting the study on protein catalytic function. The accuracy of the molecular modeling predictions was corroborated by the results of the in vitro enzyme reaction assays [48,49]. The expression level of *NgF3H*, situated upstream of dihydromyricetin, exhibited no correlation with the dihydromyricetin content. This observation inferred that *NgF3′5′H3* served as the pivotal node gene within the dihydromyricetin synthesis pathway in the bud tips of *N. grossedentata*. It can bind effectively and specifically to the precursor metabolites of dihydromyricetin, thereby facilitating the conversion of these precursor metabolites into dihydromyricetin. Elevated expression of the NgMYB71 transcriptional regulator in bud tips activated a corresponding increase in the expression of *NgF3′5′H3* within the same region. The NgF3′5′H3 specifically interacted with dihydromyricetin precursor metabolites, thereby facilitating the numerous conversion of taxifolin to dihydromyricetin in *N. grossedentata*. Consequently, this process resulted in a substantial increase in dihydromyricetin content in the bud tips of *N. grossedentata* compared to that in other tissues or other plants.

## 4. Materials and Methods

### 4.1. Plant Materials

Fifteen germplasms of *Nekemias grossedentata*, each with a consistent growth period of 4 to 5 years, were selected from the variety garden situated in Yongshun County, Xiangxi Tujia and Miao Autonomous Prefecture, Hunan Province (29°28′ N, 109°88′ E, 472 m). *Nekemias grossedentata* exhibits new bud tip growth from April to October. Consequently, in this study, bud tips from various germplasms growing in spring, summer, and autumn were collected in April, August, and October as research samples.

### 4.2. Quantification of Total Flavonoids and Dihydromyricetin in the Bud Tip of Nekemias grossedentata

Numerous studies have indicated that flavonoids exhibit greater stability at temperatures below 80 °C [50,51]. Most studies have indicated that the structural properties of dihydromyricetin are more stable at temperatures below 100 °C [52,53]. The collected bud tips were stored and transported at room temperature, then subjected to a drying process in an oven set at 60 °C. The dried material was then ground using a 60-mesh sieve, sealed, and stored at 4 °C for subsequent extraction and quantification of total flavonoids and dihydromyricetin.

Total flavonoids were extracted from the bud tips using ultrasound-assisted ethanol extraction (60 °C, 30 min, 100 Hz). The total flavonoid content of the extract was quantified using ultraviolet (UV) spectrophotometry [54].

In this study, dihydromyricetin was extracted from bud tips using ultrasound-assisted ethanol extraction (60 °C, 30 min, 100 Hz). The extract was subsequently diluted 500-fold with 80% methanol solution, filtered through a 0.22 μm sterile membrane, and analyzed for dihydromyricetin content in bud tip samples using high-performance liquid chromatography (HPLC) [38]. The chromatographic column used was a C18 analytical column (5 μm, 4.6 × 150 mm). The detection wavelength was set at 290 nm, and the column temperature was consistently maintained at 30 °C. An injection volume of 10 μL was used, with a detection duration of 25 min. The mobile phase A comprised 0.1% aqueous phosphoric acid, and the mobile phase B comprised acetonitrile. An isocratic elution method was utilized, with flow rates set at 0.85 mL/min for mobile phase A and 0.15 mL/min for mobile phase B.

### 4.3. Collection, Extraction and Detection of Targeted Flavonoid Metabolome Samples

Fresh bud tips of PZY001 and PZY004 germplasms were collected as study materials in April, August, and October, rinsed with sterile water, promptly placed in liquid nitrogen for rapid freezing, and transported to Wuhan Meteville Biotechnology Co (Wuhan, China). Following vacuum freeze-drying, the bud tip samples were ground using a ball mill at room temperature until powdered (30 Hz, 1.5 min). Subsequently, 20 mg of the sample was weighed and added to 10 μL of an internal standard mixing solution with a concentration of 4000 nmol/L, along with 500 μL of a 70% methanol solution. The sample was sonicated for 10 min, followed by centrifugation at 4 °C and 12,000 rpm for 5 min. The supernatant was filtered through a 0.22 μm filter. A 10 μL volume of the sample solution was combined with 990 μL of the extractant. On this basis, 10 μL was further added to 990 μL of the extractant. The diluted extract was qualitatively and quantitatively analyzed using ultra-performance liquid chromatography–tandem mass spectrometry (UPLC-MS/MS) [55]. The samples were categorized into six groups: PZY001: ST-1-4, ST-1-8, and ST-1-10; PZY004: ST-4-4, ST-4-8, and ST-4-10. Each group was analyzed with four biological replicates.

### 4.4. Evaluation and Analysis of Metabolomic Data Results

Using the detection data for each standard, the data obtained through mass spectrometry were subjected to qualitative analysis. In the Multiple Reaction Monitoring (MRM) mode of triple quadrupole mass spectrometry, the quadrupole initially screened the precursor ions of the target substances to eliminate interference from ions associated with other molecular weight substances. The precursor ions were then ionized in a collision chamber, leading to the formation of multiple fragments. These fragment ions were subsequently filtered through a triple quadrupole to select the desired characteristic fragment ions and exclude non-target ions. Following the acquisition of mass spectrometry data from various samples, the integrated peak areas of all detected target compounds were used to calculate their contents based on a standard curve.

Following the application of unit variance scaling to the raw quantitative metabolite data from the six groups of bud tip samples, principal component analysis (PCA) was conducted using the statistical function prcomp in R software (version 3.5.1). Z-score standardization was performed based on the mean and standard deviation of the original dataset. Metabolite quantification data for the six groups of bud tip samples were analyzed using orthogonal partial least squares-discriminant analysis (OPLS-DA). The reliability of the developed model was confirmed through the metrics R^2^X, R^2^Y, and Q^2^, with the general requirement that R^2^ and Q^2^ exceed 0.4. The variable importance in projection (VIP) values obtained from the OPLS-DA analysis were combined with *p*-values from univariate statistical *t*-tests to identify significantly different metabolites between the comparison groups using the criteria of VIP ≥ 1 and *p* < 0.05. Following Z-score normalization of the quantitative metabolite data from the six groups of bud tip samples, heatmaps were generated using the R software ComplexHeatmap package, and hierarchical clustering analyses were conducted to examine the accumulation patterns of metabolites in different samples [56].

### 4.5. Transcriptome Sequencing

Significant variations were observed in the dihydromyricetin content within the bud tips of the PZY004 germplasm across three distinct periods. Consequently, fresh bud tips of the PZY004 germplasm, collected in April, August, and October, were used as study materials. These samples were rinsed with sterile water post-harvest, promptly immersed in liquid nitrogen for rapid freezing, and subsequently placed on dry ice for transportation to Suzhou Panomic Biomedical Technology Co. (Suzhou, China) for transcriptome sequencing. The three groups of bud tip samples were named ST-4-4, ST-4-8, and ST-4-10, and four biological replicates were set for each group.

Next-generation sequencing (NGS) technology was employed using the Illumina platform to sequence the eukaryotic reference transcriptome of the samples. RNA was extracted from the bud tips using the Invitrogen TRIzol kit (Thermo Scientific, Waltham, MA, USA). Following the acquisition of raw sequencing data, the data were filtered to yield clean data. Based on the genome data of *Nekemias grossedentata* measured in the previous period of our research group, the *Nekemias grossedentata* genome data served as the reference genome, and HISAT2 software (https://daehwankimlab.github.io/hisat2/, accessed on 18 May 2025) was used to align the sequencing results with the reference genome for quality assessment. The genes and transcripts obtained post-transcriptome assembly were compared with the NR, Swiss-Prot, EggNOG, GO, and KEGG databases. Gene transcript levels were quantified as fragments per kilobase per million fragments (FPKM) values.

### 4.6. Screening of Differentially Expressed Genes

The DESeq2 analysis program was used to examine variations in gene expression levels between the sample groups. Differentially expressed genes (DEGs) between the two groups of samples were identified using the criteria |log2(FoldChange)| ≥ 1 and *p* < 0.05.

### 4.7. Trend Clustering Analysis

Genes from three temporally continuous sample groups, ST-4-4, ST-4-8, and ST-4-10, were categorized based on distinct expression patterns utilizing the STEM Trend Cluster Analysis (Series Test of Cluster) method. The selection criteria included |log2(FoldChange)| ≥ 1, with eight trends identified. The expression of genes enriched in each trend was analyzed for significant differences among the three periods. Expression trends exhibiting similar changes in metabolite content were subsequently selected for further screening of candidate core genes.

### 4.8. Integrative Analysis of Transcriptome and Metabolome to Identify Candidate Core Genes

Gene Ontology (GO) enrichment analysis and Kyoto Encyclopedia of Genes and Genomes (KEGG) pathway enrichment analysis were conducted on the differentially expressed genes within the selected trends that were similar to the alterations in dihydromyricetin content. This was performed to predict and visualize the potential functions of the genes within these trends and identify the metabolic pathways that may be enriched.

Structural genes and transcription factors involved in the dihydromyricetin synthesis pathway were manually screened from the three selected profiles. The expression levels of these genes were correlated with the contents of the significantly different metabolites identified in this pathway to identify potential core candidate genes.

### 4.9. Co-Expression Network Analysis of Genes

The relationship between the expression of selected candidate core structural genes and candidate transcription factors was examined, and the gene co-expression network was constructed using Cytoscape software (version 3.9.1). Additionally, the Maximum Clique Centrality algorithm was employed to identify candidate transcription factors that exhibited significant associations with candidate core structural genes.

### 4.10. Quantitative Real-Time PCR

The expression levels of two candidate core structural genes and eight candidate transcription factors were quantified in the bud tips of PZY004 germplasm in April, August, and October, as well as in roots, stems, mature leaves, and bud tips in October of the PZY004 germplasm, using qRT-PCR.

Primer Premier 5 software was employed to design specific upstream and downstream primers for each gene (Table S6), with the *GAPDH* gene serving as the internal reference gene [57]. The ReverAid First Strand cDNA Synthesis Kit (Thermo Scientific, Waltham, MA, USA) was used to synthesize cDNA. A 5-fold dilution was utilized for qRT-PCR experiments. The reaction system was configured using the SYBR Green Pro Taq HS Premixed qPCR kit (with high ROX) (Accurate Biology, Changsha, China). Subsequently, the reaction system was analyzed using a StepOne TM Real-Time PCR instrument (Accurate Biology, Changsha, China). The expression levels of each gene in the samples were quantified utilizing the 2^−ΔΔCT^ method.

### 4.11. Molecular Cloning and Bioinformatics Analysis

The conserved structural domains in proteins were predicted using NCBI CD-Search and InterPro. The physicochemical properties of the proteins were analyzed using ProtParam. Signal peptide prediction was performed using SignalP-6.0, and transmembrane structural domains were predicted using DeepTMHMM-1.0. Secondary structure prediction was performed using SOPMA.

MEGA 11 software was used to construct a phylogenetic tree between *NgF3′5′H3* and *F3′5′H*, utilizing sequences obtained from the NCBI database. Multiple sequence alignments were conducted using the ESPript 3 analysis platform to identify conserved amino acid sites. The three-dimensional structure of taxifolin was retrieved from the PubChem database. Homology modeling of NgF3′5′H3 was performed using the SWISS-MODEL. The binding interaction between NgF3′5′H3 and taxifolin was predicted using AutoDock Vina software (version 1.5.7). PyMOL software (version 3.1.0) was employed to visualize the interaction between NgF3′5′H3 and taxifolin.

## 5. Conclusions

In this study, flavonoids in *N. grossedentata* bud tips were qualitatively and quantitatively assessed using a targeted flavonoid metabolome technique. A total of 65 flavonoid metabolites were detected, with dihydromyricetin (DHM) exhibiting the highest content (37.5%) across all groups of bud tip samples as the predominant flavonoid, followed by myricetin (0.144%) and taxifolin (0.141%). Through integrated transcriptomic and metabolomic analyses, this study identified that only *NgF3′5′H3* expression showed a significant positive correlation with DHM content. Co-expression analysis and qRT-PCR validation showed that only NgMYB71 exhibited a significant positive correlation with *NgF3′5′H3* expression, with a consistent trend across the three periods and four tissues. Consequently, *NgF3′5′H3* and *NgMYB71* were identified as core genes influencing the substantial synthesis of DHM in *N. grossedentata*. Furthermore, qRT-PCR analysis confirmed that the candidate genes expression patterns were consistent with transcriptome sequencing results. The elevated expression of NgMYB71 in the bud tips activated the high expression of *NgF3′5′H3* in the bud tips, thereby promoting extensive dihydromyricetin synthesis in the bud tips. Molecular docking results further revealed that NgF3′5′H3 had a strong binding affinity for taxifolin. *NgF3′5′H3* was identified as a core node gene in the dihydromyricetin synthesis pathway in *N. grossedentat*. The elevated expression of *NgF3′5′H3* in bud tips, with the NgF3′5′H3 protein strongly binding to dihydromyricetin precursor metabolites, catalyzed dihydromyricetin synthesis through the extensive conversion of precursor substances. Consequently, the DHM content in *N. grossedentata* bud tips was significantly higher than that in other tissues and plants. This study provided a significant foundation for elucidating the molecular mechanisms underlying the extensive synthesis of dihydromyricetin in the bud tips of *N. grossedentata*. Furthermore, it provided a theoretical framework for enhancing dihydromyricetin production from biosynthetic sources and for the development and utilization of *Nekemias grossedentata* resources.

## Figures and Tables

**Table 1:** Candidate structural genes screened from profiles 3, 4, and 7.

Gene	Profile 3	Profile 4	Total
*F3H*	*NgF3H* (*Chr09.g12877*)		1
*F3′5′H*	*NgF3′5′H2* (*Chr04.g05287*)	*NgF3′5′H3* (*Chr04.g05289*)	2
*F* *LS*	*NgFLS3* (*Chr01.g01918*) *NgFLS4* (*Chr01.g01919*) *NgFLS5* (*Chr01.g01921*)		3

**Table 2:** Correlation analysis of candidate core structural genes and candidate transcription factors expression.

Name	*NgF3′5′H3*	*NgFLS5*
NgMYB71	0.9514 **	0.0558
NgWRKY24	−0.5756	−0.4216
NgWRKY37	−0.5095	−0.3103
NgWRKY47	0.1440	0.0804
NgbHLH50	0.2254	−0.6826
NgbHLH83	−0.0641	0.1485
NgbHLH125	−0.2197	−0.2630
NgbHLH128	0.0470	0.8500 *

Note: ** Significant correlation at 0.01 level; * Significant correlation at 0.05 level.

## Data Availability

Data are contained within the article and Supplementary Materials.

## Supplementary Materials

The following supporting information can be downloaded at https://www.mdpi.com/article/10.3390/plants14101561/s1. Figure S1: Trend cluster analysis of differentially expressed genes. ST-4-4: PZY004 germplasm bud tips in April; ST-4-8: PZY004 germplasm bud tips in August; ST-4-10: PZY004 germplasm bud tips in October; Figure S2: Gene Ontology (GO) enrichment analysis of differentially expressed genes across three profiles. (A) Profile 3. (B) Profile 4. (C) Profile 7; Figure S3: Representation of KEGG-enriched pathways associated with the differentially expressed genes across the three profiles. (A) Profile 3. (B) Profile 4. (C) Profile 7; Figure S4: Analysis of conserved domain of amino acid sequence of NgF3′5′H3; Figure S5: Phylogenetic analysis of the NgF3′5′H3 gene and F3′5′H genes across multiple species; Figure S6: Sequence comparison of conserved SRS6 motifs in NgF3′5′H3. The box indicates the amino acid sites responsible for hydroxylation; Figure S7: Analysis of cis-acting elements of 2k bp promoter upstream of NgF3′5′H3 gene. (A) Visualization and analysis of four types of cis-acting elements. (B) Prediction of the types and quantities of development response elements, hormone response elements, and stress response elements within the promoter of NgF3′5′H3; Table S1: The all 65 metabolites; Table S2: Screening results of differential metabolites between different comparison groups; Table S3: Quality control analysis of transcriptome sequencing data; Table S4: The transcriptome sequencing data were compared with the reference genome; Table S5: Correlation analysis between structural genes and significantly different metabolites; Table S6: Primers for qRT-PCR.
