# Supplementary material for: Integrated Transcriptomic and Targeted Metabolomic Analyses Elucidate the Molecular Mechanism Underlying Dihydromyricetin Synthesis in Nekemias grossedentata
Source: Plants (Basel). 2025 May 21;14(10):1561. doi: 10.3390/plants14101561 (PMC12115215; doi:10.3390/plants14101561)
Supplement: Supplementary file 1 [file plants-14-01561-s001.zip › Supplementary figures.pdf]

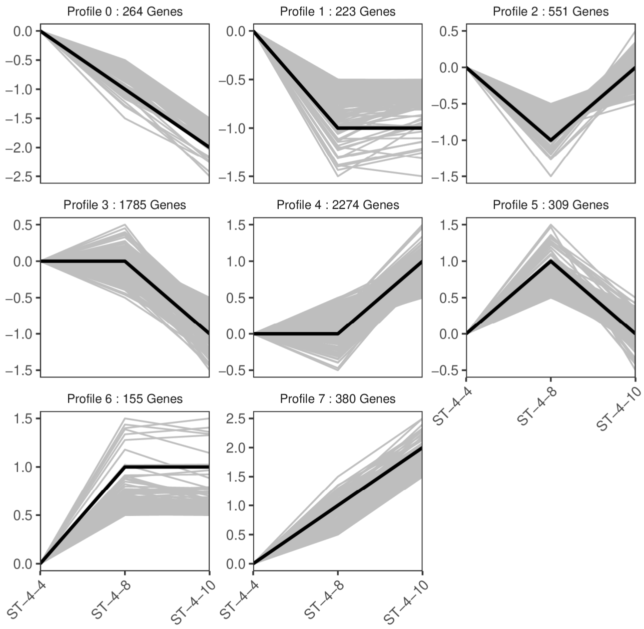

Figure S1: Trend cluster analysis of differentially expressed genes. ST-4-4: PZY004 germplasm bud tips in April; ST-4-8: PZY004 germplasm bud tips in August; ST-4-10: PZY004 germplasm bud tips in October.

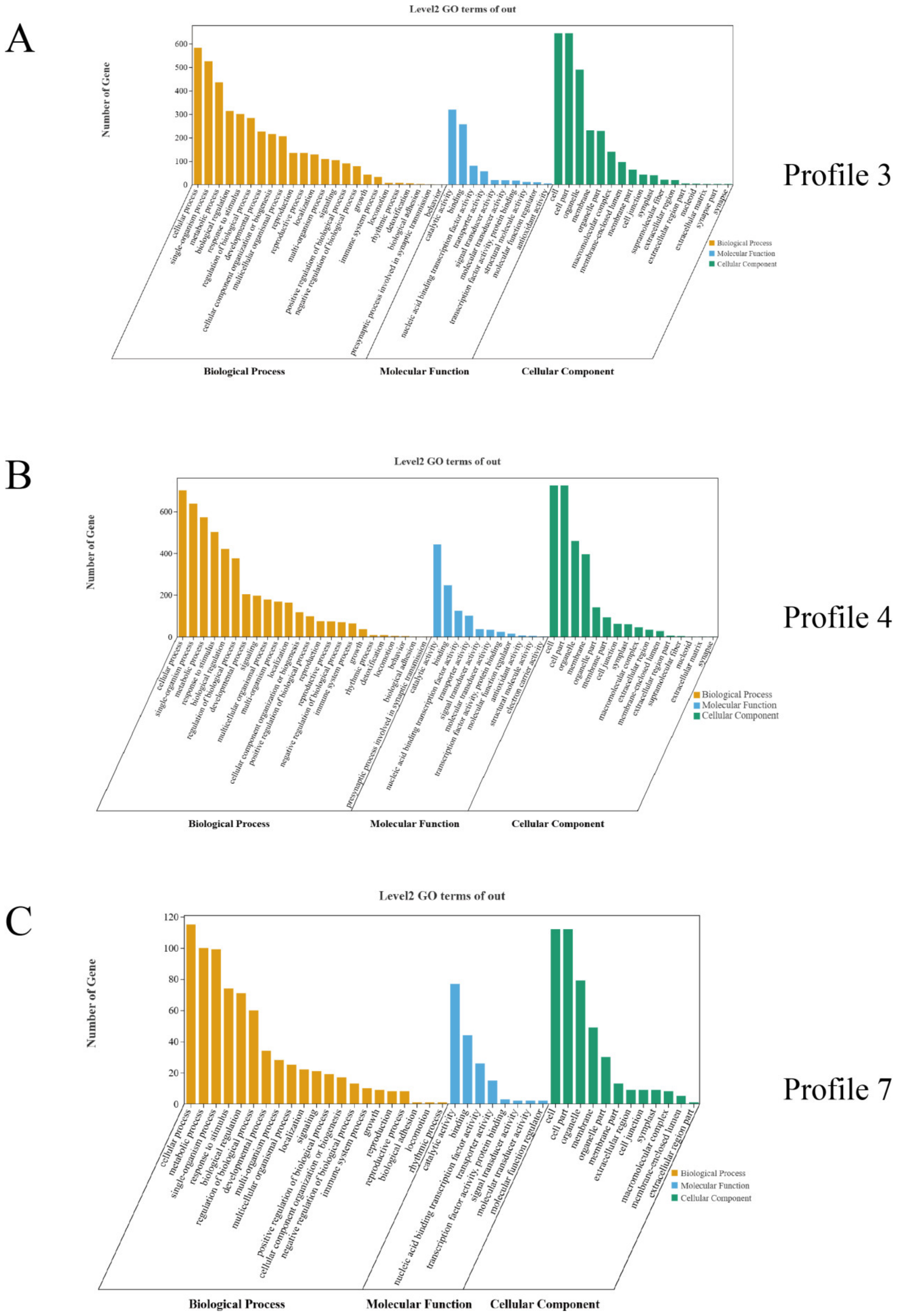

Figure S2: Gene Ontology (GO) enrichment analysis of differentially expressed genes across three profiles. (A) Profile 3. (B) Profile 4. (C) Profile 7.

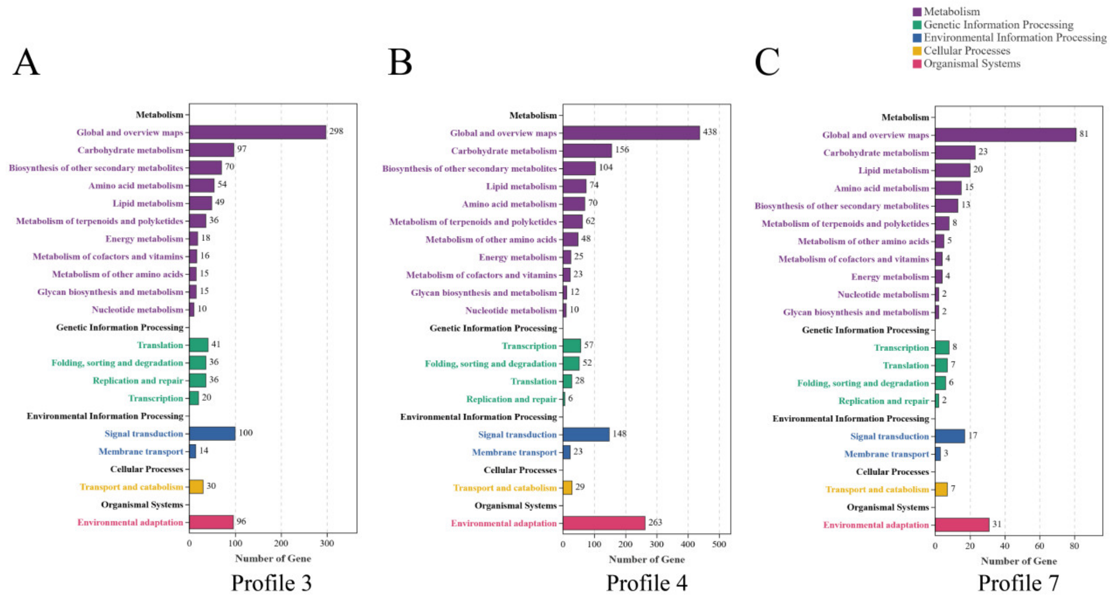

Figure S3: Representation of KEGG-enriched pathways associated with the differentially expressed genes across the three profiles. (A) Profile 3. (B) Profile 4. (C) Profile 7.

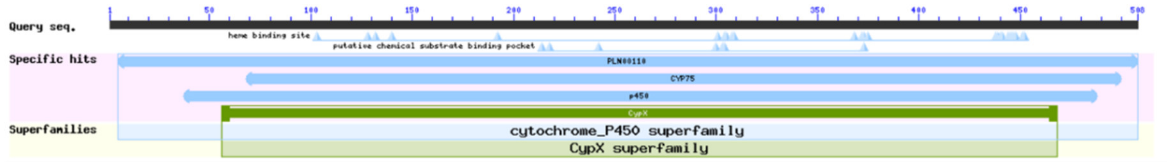

Figure S4: Analysis of conserved domain of amino acid sequence of NgF3′5′H3.

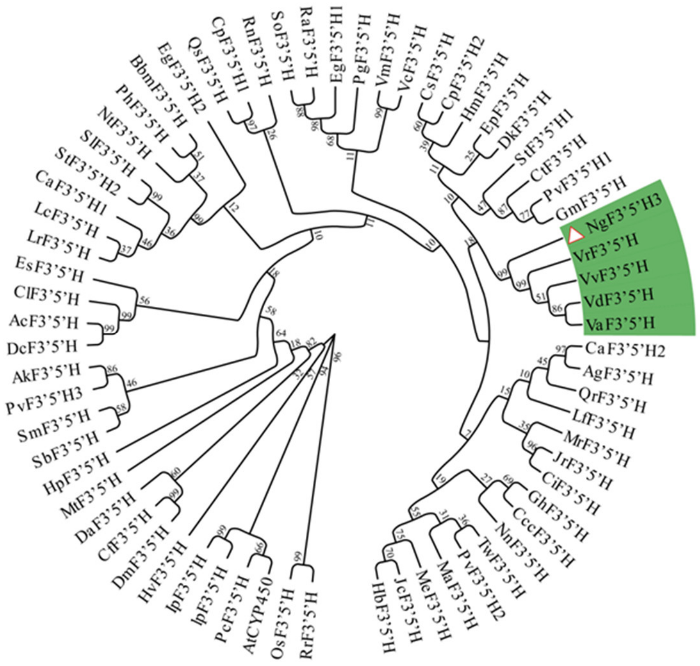

Figure S5: Phylogenetic analysis of the *NgF3′5′H3* gene and *F3′5′H* genes across multiple species.

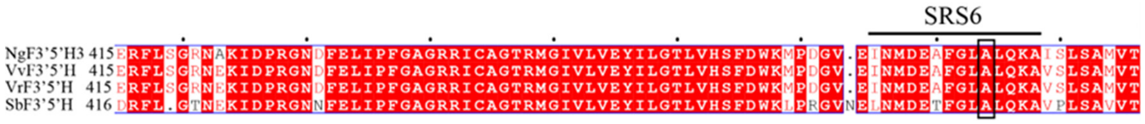

Figure S6: Sequence comparison of conserved SRS6 motifs in NgF3'5'H3. The box indicates the amino acid sites responsible for hydroxylation.

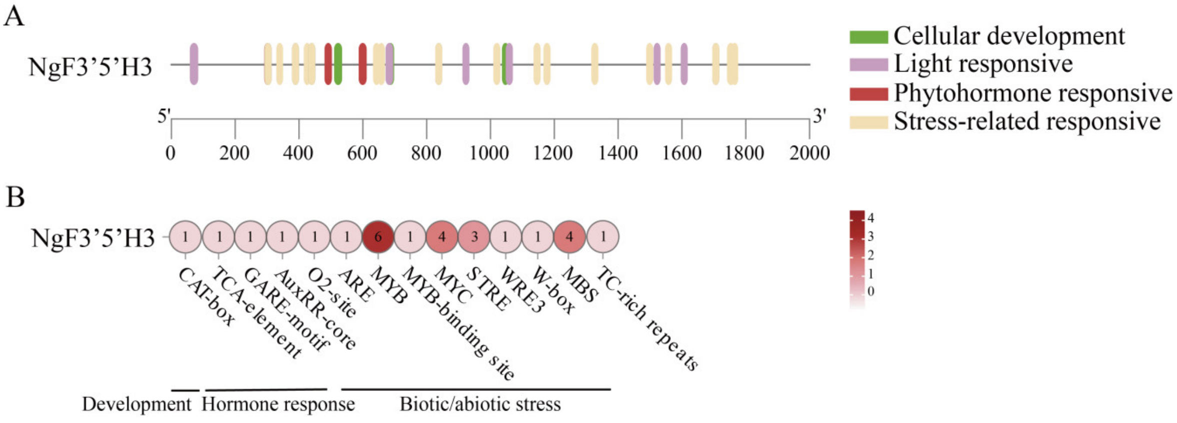

Figure S7: Analysis of cis-acting elements of 2k bp promoter upstream of *NgF3′5′H3* gene. (A)Visualization and analysis of four types of cis-acting elements. (B)Prediction of the types and quantities of development response elements, hormone response elements, and stress response elements within the promoter of *NgF3′5′H3*.
